# Low Dose CT Perfusion With K-Space Weighted Image Average (KWIA)

**DOI:** 10.1109/TMI.2020.3006461

**Published:** 2020-11-30

**Authors:** Chenyang Zhao, Thomas Martin, Xingfeng Shao, Jeffry R. Alger, Vinay Duddalwar, Danny J. J. Wang

**Affiliations:** Keck School of Medicine, Stevens Neuroimaging and Informatics Institute, University of Southern California, Los Angeles, CA 90033 USA; Mays Cancer Center, Radiation Oncology, University of Texas Health Science Center, San Antonio, TX 78229 USA, and also with Hura Imaging, Inc., Calabasas, CA 91302 USA; Keck School of Medicine, Stevens Neuroimaging and Informatics Institute, University of Southern California, Los Angeles, CA 90033 USA; Department of Neurology, University of California at Los Angeles, Los Angeles, CA 90095 USA, also with the Advanced Imaging Research Center, University of Texas Southwestern Medical Center, Dallas, TX 75390 USA, and also with Hura Imaging, Inc., Calabasas, CA 91302 USA; Keck School of Medicine, Stevens Neuroimaging and Informatics Institute, University of Southern California, Los Angeles, CA 90033 USA; Keck School of Medicine, Stevens Neuroimaging and Informatics Institute, University of Southern California, Los Angeles, CA 90033 USA, and also with Hura Imaging, Inc., Calabasas, CA 91302 USA

**Keywords:** Perfusion imaging, X-ray imaging and computed tomography, brain, image reconstruction - analytical methods, image enhancement

## Abstract

CTP (Computed Tomography Perfusion) is widely used in clinical practice for the evaluation of cerebrovascular disorders. However, CTP involves high radiation dose (≥ ~200mGy) as the X-ray source remains continuously on during the passage of contrast media. The purpose of this study is to present a low dose CTP technique termed K-space Weighted Image Average (KWIA) using a novel projection view-shared averaging algorithm with reduced tube current. KWIA takes advantage of k-space signal property that the image contrast is primarily determined by the k-space center with low spatial frequencies and over-sampled projections. KWIA divides each 2D Fourier transform (FT) or k-space CTP data into multiple rings. The outer rings are averaged with neighboring time frames to achieve adequate signal-to-noiseratio (SNR), while the center region of k-space remains unchanged to preserve high temporal resolution. Reduced dose sinogram data were simulated by adding Poisson distributed noise with zero mean on digital phantom and clinical CTP scans. A physical CTP phantom study was also performed with different X-ray tube currents. The sinogram data with simulated and real low doses were then reconstructed with KWIA, and compared with those reconstructed by standard filtered back projection (FBP) and simultaneous algebraic reconstruction with regularization of total variation (SART-TV). Evaluation of image quality and perfusion metrics using parameters including SNR, CNR (contrast-to-noise ratio), AUC (area-under-the-curve), and CBF (cerebral blood flow) demonstrated that KWIA is able to preserve the image quality, spatial and temporal resolution, as well as the accuracy of perfusion quantification of CTP scans with considerable (50–75%) dose-savings.

## I. Introduction

**C**OMPUTED Tomography Perfusion (CTP) of the brain is a widely used imaging technique that provides assessments of regional blood supply, and hemodynamic information to distinguish the ischemic core from penumbral tissue, helping with decision making for recanalization therapy in cerebral ischemia [1]–[4]. In a typical CTP scan, a dataset of time-resolved CT images is acquired over the scan duration (223C 1 min) to track the passage of the contrast bolus through the intracranial vasculature. The contrast enhancement of the tissue over time is depicted by the time density curve (TDC), and multiple perfusion parameters such as cerebral blood flow (CBF), cerebral blood volume (CBV), mean transit time (MTT), can be derived from the TDC information [5]. The repeated CT scans that are performed on the same brain region during the passage of a contrast bolus resulting in a high radiation dose to patients. For example, with a typical clinical setting of CTP scan acquisition parameters using a tube voltage of 80 keV, tube current of 150 mAs, and temporal sampling rate of 1 image/2s according to the ALARA (As Low As Reasonably Achievable) principle, the resultant dose can be about 200 mGy which is approximately 3 times higher than that of a standard head CT [6].

Recently, several techniques have been applied for radiation dose reduction in CTP scans, including reduction of tube current and/or tube voltage, as well as the use of noise reduction techniques such as iterative reconstruction (IR) [7], [8]. Typical IR methods include the adaptive statistical iterative reconstruction (ASIR) [9], and model-based iterative reconstruction (MBIR) [10]. However, IR methods often yield blotchy image appearance and longer computational time [11]. Although the application of IR in standard CT scans has been improving due to enhanced computational power, its application in CTP is very limited due to the high complexity and significant computational overhead for processing dynamic CTP image series. It is also possible to lower the radiation dose by reducing the temporal sampling frequency of CTP, however, this approach yields insufficient temporal information for accurate quantification of hemodynamic parameters [12].

During the past 3 years, deep learning (DL) techniques have been explored for CT imaging to reduce radiation dose z, such as residual neural network [14] and generative adversarial network (GAN) [16], [18], [19] based denoising. The deep networks have been expanded to incorporate iterative steps [20], [21] to improve the performance and robustness for denoising low-dose CT images. More recently, DL methods have been applied for low-dose CTP using Iterative Residual-artifact Learning net (IRLNet) [22] and Spatial-Temporal Image Restoration Net (STIR-Net) [12]. The advantages of DL techniques, as compared to existing IR methods for low-dose CT, include short computation time (nearly instantaneous once trained) and better retainment of the texture and resolution of CT images. However, DL methods are highly dependent on the training datasets which may be specific to the CT scanners and protocols used for data collection.

K-space Weighted Image Contrast (KWIC) is a technique originally used in 4D dynamic MRI with radial trajectories to shorten the scan time using sparse sampling [23]. Based on the central slice theorem, CT sinogram data can be converted to 2D Fourier space (equivalent to k-space in MRI), making it feasible for the adaptation of KWIC to CT perfusion with reduced dose through the sparse sampling of projections followed by view-sharing. In our proof-of-concept study, a specific sparse sampling scheme was employed to achieve up to 75% dose reduction while maintaining both high image quality and quantification accuracy of CTP scans [24]. However, the implementation of the KWIC algorithm requires rapid-switching pulsed X-ray at pre-specified rotation angles–a hardware capability yet to be implemented by commercial CT vendors.

The purpose of this study is to introduce a variant of the KWIC algorithm termed k-space weighted image average (KWIA) that preserves high spatial and temporal resolutions as well as image quality of low-dose CTP data (50–75% dose reduction), yielding images comparable to those of standard CTP scans. There are three major advantages and contributions of KWIA compared to existing denoising methods for low-dose CTP: 1) KWIA does not require modification of existing CT hardware, and can use standard low-dose techniques such as tube current reduction; 2) KWIA is computationally simple and fast (non-iterative), therefore doesn’t affect clinical workflow; 3) KWIA preserves the texture as well as spatial and temporal resolution of CTP images. In this paper, we first present the theoretical framework of KWIA, and demonstrate its feasibility using a digital phantom, a physical phantom, and clinical data.

## II. Theory

In a typical CT scan, the X-ray tube and detectors continuously rotate around a center point. At each specific constant angular interval, the X-ray tube emits a fan beam X-ray which will be received by an array of detectors and processed to form a fan beam projection signal. Based on the central slice theorem, 1D Fourier transform (FT) can be performed along each parallel beam projection, which can be obtained from fan beam projections after rebinning, to form a ‘k-space’ like CT data. To meet the Nyquist theory of radial sampling, the sampling rate on the periphery of this k-space should be no less than the sampling rate on each projection to avoid streaking. Thus, the number of projections in a CT scan *N*_*proj*_ , in theory, should satisfy

(1) $$N_{\text{proj}}\geq \frac{\pi }{2}N_{\text{detector}}$$

In practice, the number of projections used in a CT scan can be slightly lower than the theoretical number, since the field-of-view (FOV) of CT images is generally smaller than the width of the detector array. Assuming *R* is the radius of the radial k-space region that satisfies the Nyquist theory, it is determined by

(2) $$R=\frac{N_{\text{proj}}}{\pi }$$

**Fig. 1.**
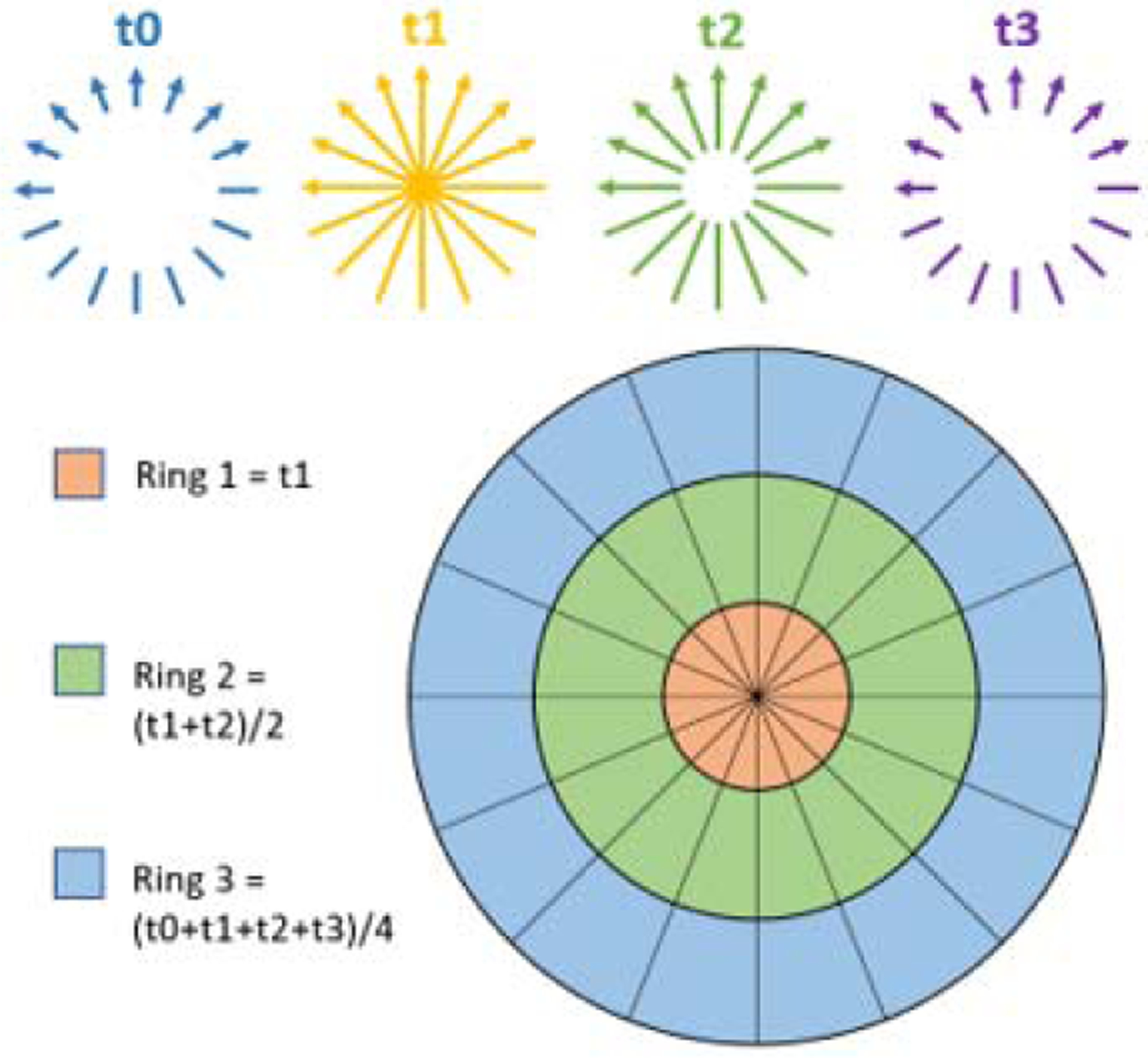
Schematic diagram of KWIA. Four time frames of CTP data (*t*_0_−*t*_3_) with reduced radiation dose are acquired. Each 2D FT or k-space can be divided into multiple rings. Outer rings can be averaged between neighboring time frames to improve SNR.

The most common practice for low dose CT includes reduction of tube current and/or tube voltage. Without loss of generality, we will focus on low dose CT with reduced tube current in this paper (tube voltage reduction will be discussed later). There is a direct proportional relationship between the applied tube current and the square root of the SNR in reconstructed CT images [25]. For example, reducing the tube current by $\frac{1}{2}$ will result in the SNR of CT images to be $\frac{\sqrt{2}}{2}$ of the original SNR. However, the effect of SNR reduction is not evenly distributed across the 2D FT or k-space. As shown in Fig. 1, the center of the k-space (Ring 1) has effectively higher SNR due to the averaging effect of higher sampling density of projections. For the outer k-space, however, the progressively sparser projections will lead to deficient SNR.

For CTP imaging, such k-space property and the time-resolved image acquisition can be exploited for reducing radiation dose. Here we introduce a new algorithm termed k-space weighted image average (KWIA) to preserve high spatial and temporal resolutions as well as the image quality of low-dose CTP data (50–75% dose reduction). The proposed KWIA method divides each 2D FT or k-space CTP data into multiple rings. The central part of k-space (Ring 1) will directly use the data from a single time frame (e.g. *t*_1_ in Fig. 1), while outer k-space regions will be progressively averaged between neighboring time frames to increase SNR (e.g. Ring 2 will be averaged by 2 time frames *t*_1_ and *t*_2_, and Ring 3 averaged by 4 time frames *t*_0_ to *t*_3_). Since the image contrast is primarily determined by the central k-space region, KWIA can preserve the high effective temporal resolution of low dose CTP while maintaining high SNR and spatial resolution by view-sharing in the outer k-space regions.

The KWIA reconstructed k-space data, *S*, can be expressed by the following equation

(3) $$S_{i,k}=\sum_{d=\left\lceil -\frac{M-1}{2}\right\rceil }^{\left\lceil \frac{M-1}{2}\right\rceil }W_{d,k}S_{i+d,k}$$

where *i* is the image time frame, *k* is the distance from the k-space center, *M* is the averaging window size, and *W*_*d,k*_ is the weighting function. Note the averaging window shifts at the beginning and end of CTP time series to keep all averaged images within range. The k-space is divided into discrete rings and moving average is applied to each ring accordingly. As a proof-of-concept study, we used a short averaging window size of 1, 2, 4 for ring 1, 2, 3 respectively (based on the original KWIC algorithm) to minimize potential temporal blurring. Alternative window sizes and weighting functions will be discussed below.

Since the number of received X-ray photons *N*_*d*_, on a detector, can be estimated as the Poisson distribution of the number of incident photons (see section 3. B for detail), which is the number of emitted photons *N*_*e*_ after attenuation, SNR can be derived as follows.

(4) $$\text{SNR}=\frac{\text{Mean}}{SD}=\frac{N_{e}e^{-l_{i}}}{\sqrt{N_{e}e^{-l_{i}}}}=\sqrt{N_{e}e^{-l_{i}}}$$

Thus, for a given *N*_*e*_ and amount of attenuation, the SNR is proportional to the square root of the size of a detector, namely resolution. Because of the inverse relation between resolution and k-space coverage, SNR is inversely proportional to the radius of a k-space region.

In order to compensate for the SNR loss using projection data from low-dose CT, we can utilize a smaller center k-space region to achieve adequate SNR, and the radius of Ring 1 (*R*_1_) can be derived from Eq. 5:

(5) $$R_{1}=\frac{N_{\text{proj}}}{\pi }rSNR$$

where rSNR is the relative SNR of low dose CTP versus the full dose scan. The rest of k-space can be subsequently divided into rings that will be progressively averaged between neighboring time frames to increase SNR. The radius of Ring *n* or *R*_*n*_ can be derived from Eq. 6:

(6) $$R_{n}=R_{1}+\frac{\frac{N_{\text{detector}s}}{2}-R_{1}}{N_{\text{rings}}-1}\left(n-1\right)$$

where *N*_*rings*_ is the total number of rings, *N*_*detectors*_ is the number of detectors, and *R*_*n*_ is the derived radius for the *n*th ring. In practice, the optimal number of rings and their respective sizes can be determined empirically. The more rings that are used, higher the SNR. However, the resultant images could potentially be more susceptible to motion as well as temporal smoothing (of fine structures) between time frames. After applying KWIA, the k-space data is regridded into Cartesian space followed by 2D inverse fast FT (FFT) to generate CT images.

## III. Material and Methods

### A. KWIA Algorithm Implementation

The KWIA algorithm was implemented in Matlab (The Mathworks Inc., Natick, MA, USA) and included 5 steps: 1) performing 1D FFT of parallel beam projections along the detector row direction; 2) multiplication with a KWIA filter that separates and weighs projections into sub-apertures or rings; 3) stacking of KWIA filtered projections into a radial k-space; 4) compensating for the weighting of radial data using the VORONOI algorithm; 5) regridding of radial k-space into 2D Cartesian k-space; and finally 6) performing 2D inverse FFT of the regridded k-space data into 2D images. In step 1, the parallel beam CT projections were simulated from CTP images using the ASTRA toolbox [26], [27] for digital phantom and clinical data, while, for real scan of the phantom, fan beam projections were acquired and then rebinned into parallel beam projections. The VORONOI algorithm in step 4 was used as an efficient and accurate estimation of the density compensation for radial sampled k-space data [28]. Even though the density weights along each radial projection in k-space is just a ramp function, the VORONOI algorithm provides more flexibility for potential implementation of KWIA in more complicated CT geometries, such as 3D cone beam CT (CBCT). For the regridding algorithm of step 5, we chose the Kaiser-Bessel kernel with *β* = 16.25, window width = 7, and oversampling rate = 2 as the convolution kernel to achieve the optimal balance between side-lobe suppression and computation time [29]. Compiled Matlab programs and a sample CTP dataset can be downloaded (https://loft-lab.org/index-5.html).

### B. Digital Dynamic Phantom Simulation

A FORBILD digital phantom [30] with three time-varying vessels inserted (10, 5 and 2.5 mm in diameter respectively) was created to simulate a dynamic CT scan. Scanning parameters of the simulation are shown in Table I, and ring sizes used for KWIA reconstruction are listed in Table II. A baseline Poisson noise with an emitting X-ray photon number of 4.8 × 10^6^, which is the same value estimated for clinical CTP data in section 3. D, was added in the projection data to simulate full dose CTP scan. Accordingly, 50% and 25% dose CTP scans were generated using 2.4×10^6^ and 1.2×10^6^ as the emitting X-ray photon number, respectively. In addition, the temporal variation of vessel signals followed a pre-defined gamma variate function [31] (Eq. 7), where *C(t)* is the vessel signal, *C*_0_ is a constant which was set to 1, *t* refers to time and *α* is a parameter determining the signal changing rate (*α* = 11). The digital phantom simulation used the same scan time (54 seconds) and number of frames (27 frames) as our clinical data. The peak vessel signal was set to appear at the 10th time frame.

(7) $$C\left(t\right)=C_{0}t^{\alpha }e^{\alpha \left(1-t\right)}$$

For the simulation of low dose CTP using digital phantom, we hypothesized that: (1) True compound Poisson process can be well-estimated as Poisson distribution; (2) Electronic noise, which is Gaussian distributed, can be ignored [32]–[34]. Thus, the noise model can be simplified as that, on a detector, the detected number of photons follows the Poisson distribution of the number of received photons, which is attenuated from the number of emitting photons while penetrating the body. The reduction of tube current will proportionally reduce the emitted photons. As a result, the detected number of photons by a detector under the reduced tube current can be determined by Eq. 8 [35], where *N*_*d*_ is the detected photon number, *N*_*e*_ is the emitted photon number, *l*_*i*_ is the line integral of attenuation coefficients corresponding to the detector *i*, *α* and *β* represent the full dose tube current and reduced tube current respectively.

(8) $$N_{d}=\text{Poisson}\left(\frac{\beta }{\alpha }N_{e}e^{-l_{i}}\right)$$

**Fig. 2.**
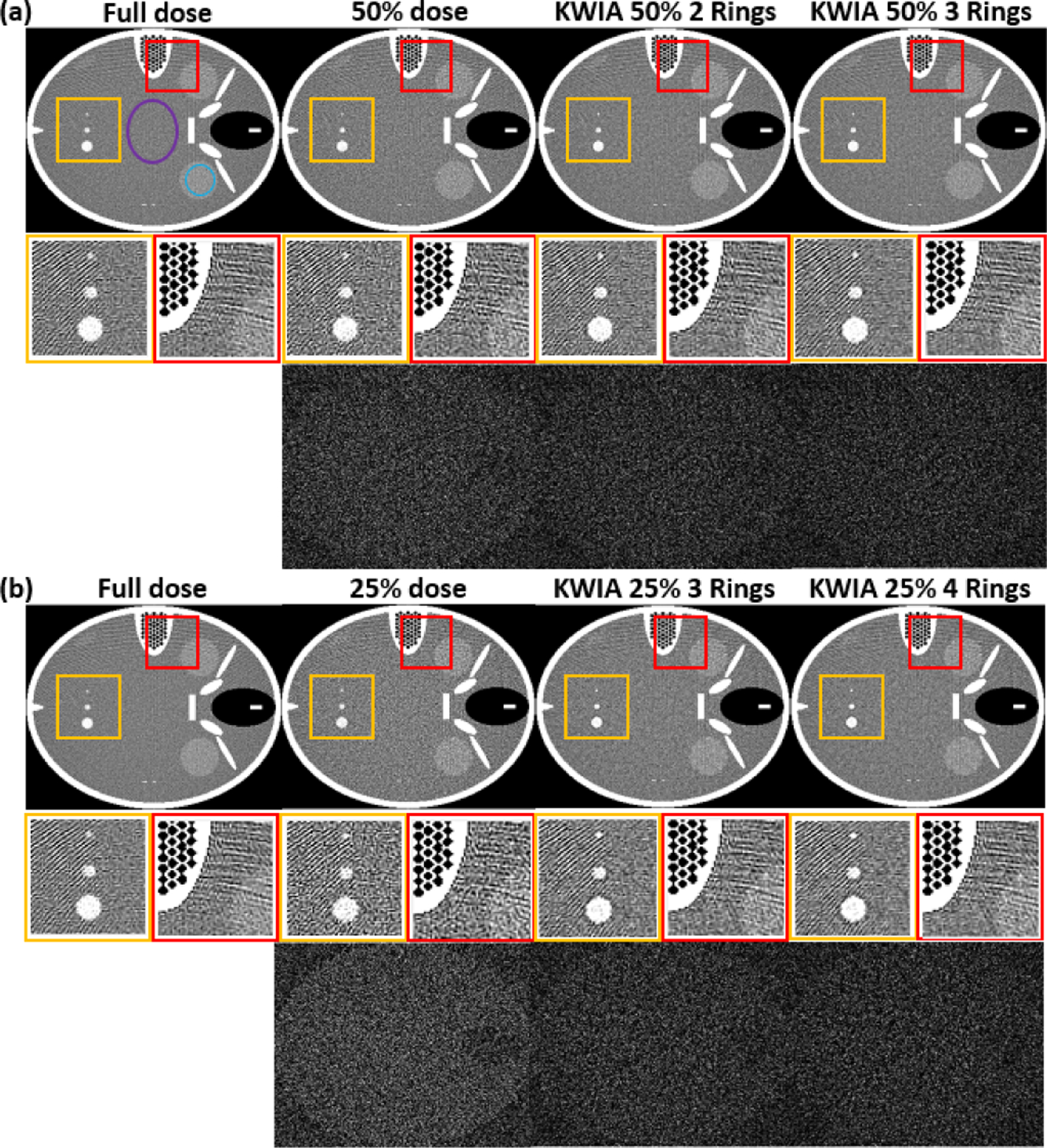
FORBILD CT phantom with 3 vessels of different sizes. (a) and (b) contains the full dose, low dose simulation, and 4 KWIA simulation results. Two ROI were enlarged to emphasize SNR change. And subtraction images (window level and window center were adjusted for visual observation) were made to show the structural change.

CTP scans with full dose, 50% dose, and 25% dose were simulated accordingly. KWIA with 2 and 3 rings were then applied to recover SNR of 50% dose CTP scans, while KWIA with 3 and 4 rings were applied to recover SNR of 25% dose CTP scans. To evaluate the performance of KWIA, the mean signal and standard deviation (SD) of noise were measured in a relatively uniform region to estimate SNR (blue circle in Fig. 2 (a)). Contrast-to-noise ratio (CNR) was also estimated using two regions (blue circle and purple circle in Fig. 2 (a)) with different mean values. The value of CNR was defined as the ratio of the difference of mean signals between two regions to the square root of the sum of their variance. To evaluate the impact of different vessel sizes on the temporal fidelity of KWIA reconstruction, the region containing 3 time-varying vessels was selected as an ROI to measure the time course, as well as temporal parameters, including area-under-the-curve (AUC), full width at half maximum (FWHM), and root mean squared error (RMSE). To better demonstrate the SNR change, subtraction images between a noiseless phantom image and simulated low dose CTP and KWIA images were generated and presented.

### C. Physical CTP Phantom Scan

The purpose of the physical phantom study was to verify the low dose simulation method, the SNR dependency of perfusion metrics, and the feasibility of KWIA reconstruction on real low dose CTP scans. A commercial CT perfusion phantom (GAMMEX, Middleton, WI, USA) was scanned on a Siemens SOMATOM Definition AS scanner with a fixed tube voltage of 100 kVp and 3 different tube currents at 200, 120 and 60 mAs respectively. The GAMMEX CTP phantom consisted of a homogenous scan disk (the imaged object shown in Fig. 4) and 4 rods (as indicated by the yellow arrows in Fig. 4), including an “artery”, a “vein” and 2 identical “brain tissue” rods, that were made of several discs with variable densities. When set in motion, the 4 rods can mimic the flow of a contrast agent through a blood-tissue network over time. Therefore, the phantom was capable of simulating blood flow through an artery, a vein and two tissue regions [36]. During the scan, three slices were imaged simultaneously in a total scan duration of 39 seconds with an interval of 1 second for each time frame. After the scan, fan beam projection data collected from scanner were rebinned into parallel beam projection data for offline reconstruction with KWIA.

The scan with the highest tube current of 200 mAs was used as the full dose, while the scans with 120 and 60 mAs were treated as 60% and 30% dose respectively. KWIA with 2 and 3 rings were applied on the 60% dose scan, while KWIA with 3 and 4 rings on the 30% dose scan. For comparison, full and low dose images were also reconstructed with standard regridding reconstruction without KWIA. Detailed scanning parameters are listed in Table I, and the sizes of rings used in KWIA are listed in Table II.

SNR was measured in a uniform region (purple circle in Fig. 4 (a)) in the scan disk of the CTP phantom, and CNR was measured between the scan disk region and a uniform region in brain tissue (blue circle in Fig. 4 (a)). The arterial input function (AIF), venous outflow function (VOF) and tissue signal curves were measured in ROIs of the artery, vein, and brain tissue, respectively.

Quantitative CTP analysis was performed using in-house MATLAB program for deconvolution based on the singular-value decomposition (SVD) algorithm [37]. Post-processing of CTP images yielded cerebral blood flow (CBF) maps. CBF values in 2 brain tissue regions of the phantom were measured for comparison across all the reconstructed perfusion maps.

### D. Clinical CTP Data Simulation

Six clinical CTP datasets, treated as full dose cases in this study, were acquired on a Toshiba Aquilion CT scanner. Detailed parameters of the CTP scan are listed in Table I. Poisson distributed noise was added to simulate the 50% and 25% low dose cases. The KWIA algorithm with 2, 3 and 4 rings was implemented and tested on the simulated low dose CTP scans respectively using the same ring sizes as those for digital dynamic phantom simulation (parameters listed in Table II).

There were a few differences in how noise was added between the clinical data and the digital phantom. In digital phantom, Poisson noise was directly added on noiseless phantom images. Given an emitted photon number *N*_*e*_ for the full dose scan, the low dose scans can be simply simulated from noiseless phantom images using $\frac{\beta }{\alpha }N_{e}$ in Eq. 8. In clinical data, however, no noiseless images were available, which required including additional noise on top of the full dose images already containing some level of noise. Also, *N*_*e*_ of the full dose images is an unknown parameter. The simulation process can be described by Eq. 9 [38]:

(9) $$N_{d}^{\beta }=\frac{\beta }{\alpha }N_{e}^{\beta }e^{-l_{i}}+{\text{Poisson}}_{0}\left(\frac{\alpha \beta -\beta^{2}}{\alpha^{2}}N_{e}^{\alpha }e^{-l_{i}}\right)$$

where $N_{d}^{\beta }$ is the received X-ray photon number at dose *β*, $N_{e}^{\alpha }$ is the emitted photon number at dose *α*, and *Poisson*_0_ refers to the Poisson distribution with zero mean. In this case, the generated data will have the desired variance and mean of $\frac{\beta }{\alpha }N_{e}^{\alpha }$. For *N*_*e*_, we estimated it as 4.8 × 10^6^ by measuring the change of resultant SNR in reconstructed image. An accurate estimation of *N*_*e*_ should allow the SNR to decrease linearly with the square root of tube current reduction, which has been validated by our physical phantom study.

The mean signal and noise SD were measured in a uniform region (blue circle in Fig. 8 (a)) in grey matter to examine the impact of KWIA on SNR. CNR was also estimated between grey matter (blue circle in Fig. 8 (a)) and white matter (purple circle in Fig. 8 (a)). The arterial input function (AIF), venous outflow function (VOF) and tissue density signal curves were measured in ROIs of the anterior cerebral artery, posterior part of the superior sagittal sinus, and uniform grey matter region without visible vessels, respectively.

Quantitative CTP analysis was performed in the same way as the physical phantom study. The mean CBF value of the whole brain were measured for all 6 datasets for comparison across all the reconstructed perfusion maps.

### E. Comparison of KWIA With Other Reconstruction Algorithms

A comparison of KWIA with two other image reconstruction methods was performed on the clinical CTP data from the perspective of noise suppression and CBF bias reduction. The two methods included filtered back projection (FBP) with ramp filter, the standard algorithm for clinical CT, and simultaneous algebraic reconstruction regularized by total variation (SART-TV) [39], a state-of-art technique for CT denoising. The FBP was implemented using ASTRA toolbox [26], [28], and TIGRE toolbox [40] was used for SART-TV implementation. Among the 3 commonly used FBP filter functions (Ram-Lak, Hann, Shepp-Logan), the Ram-Lak filter was applied in the comparison study since low-pass windowing functions like Hann and Shepp-Logan introduce trade-offs between denoising and smoothing. Quantitative comparison including SNR in GM (blue circle in Fig. 12) and WM (purple circle in Fig. 12), CNR between GM and WM, and the whole brain CBF measurements across 6 clinical datasets was performed. To achieve performance-efficiency balance, default hyperparameter values recommended by the TIGRE toolbox were selected, including 100 as the number of iterations, 1 as the step size, 15 as the value of the parameter for regularization strength, and 50 as the number of iterations in TV regularization step.

## IV. Results

### A. Digital Dynamic Phantom Simulation

Figure 2 shows the CT images (7th time frame) of 7 experimental conditions (full dose, 50% dose, 25% dose, KWIA 50% 2 Rings, KWIA 50% 3 Rings, KWIA 25% 3 Rings, KWIA 25% 4 Rings), respectively. The insets show two zoomed ROIs to highlight the SNR changes. In these two ROIs, it can be seen that the SNR was degraded in 50% and 25% dose images compared to full dose images, which was recovered by KWIA reconstruction. In addition, the subtracted images between KWIA reconstructed images and full dose images illustrate that no structured noise pattern or texture changes were induced by KWIA reconstruction.

Table III lists the SNR values of the seven experimental conditions respectively. The SNR of 50% and 25% dose images were about 73% and 52% of that in full dose images, which are consistent with theoretical prediction. KWIA, however, was able to recover the SNR of 50% and 25% dose images to be comparable to that of the full dose images. Consistent with our prediction, increased number of rings in KWIA reconstruction led to greater SNR. The CNR results of low dose simulation are consistent with our prediction as well, and KWIA was able to recover CNR to full dose level.

**Fig. 3.**
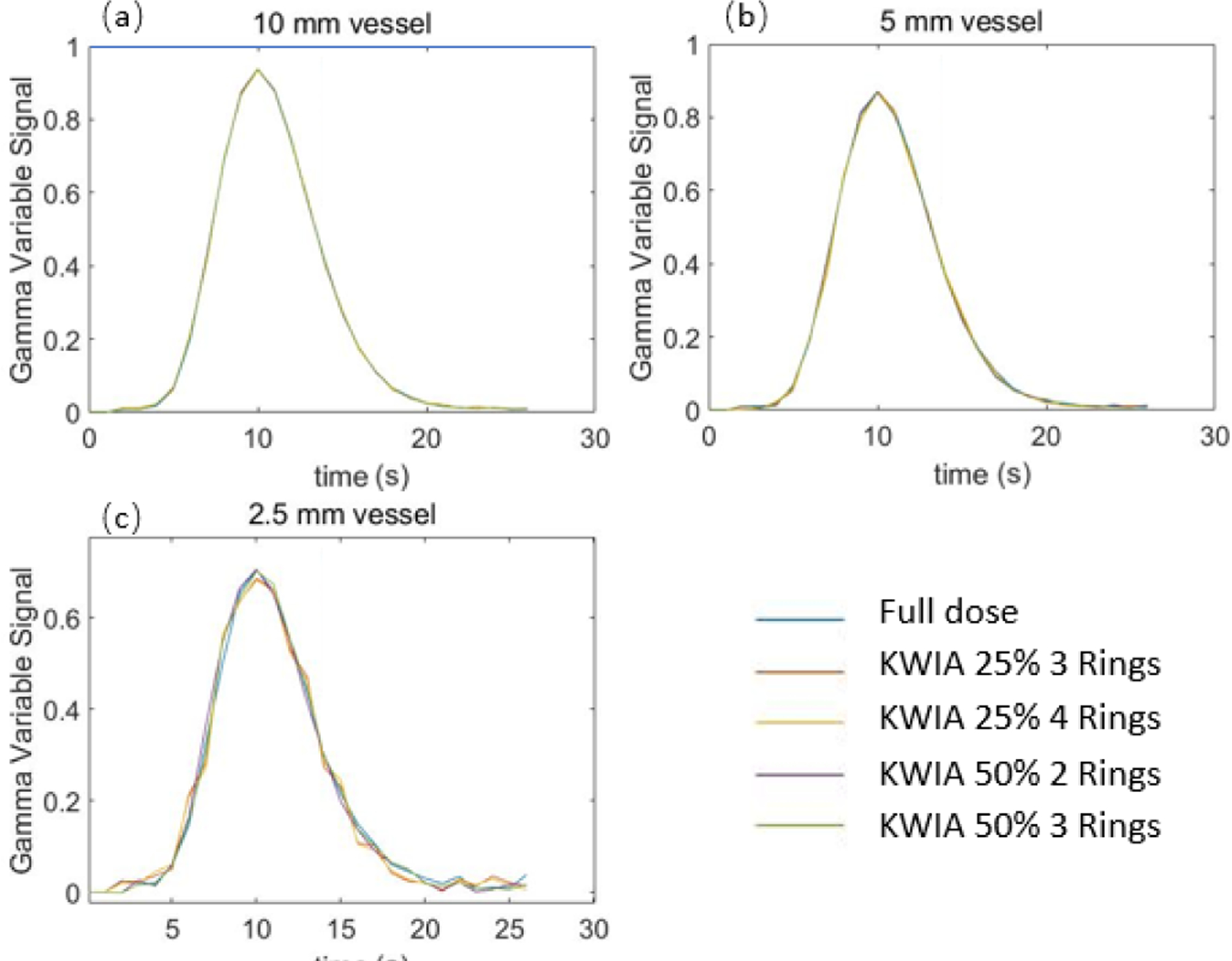
The gamma variate dynamic time curves (full dose and 4 KWIA cases) of 3 vessels with different sizes in digital phantom.

Temporal signals of the 3 vessels with different sizes (2.5, 5, and 10 mm in diameter) are shown in Fig. 3. In 10 mm (Fig. 3 (a)) and 5 mm (Fig. 3 (b)) vessels, no apparent difference between KWIA and full dose curves can be observed. This shows the capability of KWIA to preserve high temporal resolution. However, slight reduction in the maximum peak can be observed in 2.5 mm (Fig. 3 (c)) vessel of KWIA images, likely due to temporal blurring caused by the averaging of high frequency k-space data between neighboring time frames in KWIA.

To quantitatively estimate the effect of temporal blurring, temporal parameters including AUC, FWHM, and RMSE were calculated and shown in Table IV. There is up to about 1% difference in AUC and 7% difference in FWHM, respectively, for the smallest vessel. The RMSE is generally small (< 0.01) for 5 and 10 mm vessels, and increases up to 0.027 for the 2.5 mm vessel. The RMSE is smaller with higher dose and fewer rings used.

### B. Physical CTP Phantom Experiment

**Fig. 4.**
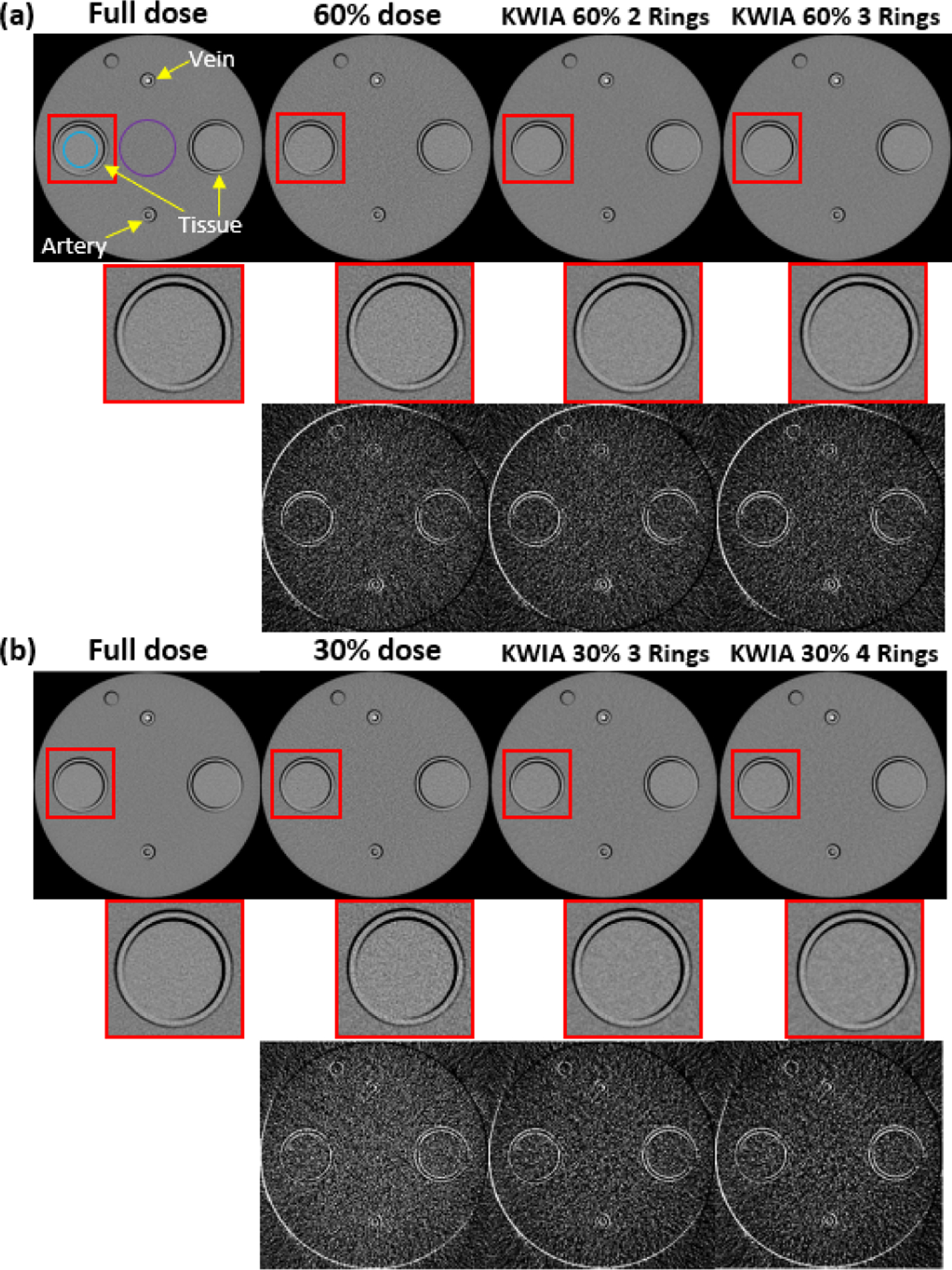
Scans of the CTP phantom. (a) and (b) contains the full dose (200 mAs), low dose (120 mAs and 60 mAs), and 4 KWIA reconstruction results. An ROI was enlarged to emphasize SNR change. And subtraction images (window level and window center were adjusted for visual observation) were made to show the structural change.

Figure 4 shows images (11th time frame) of the CTP phantom scans at a single slice within the scan disk with 7 experimental conditions, including full dose (200 mAs), 60% dose (120 mAs), 30% dose (60 mAs), 60% dose with KWIA 2 and 3 Rings, 30% dose with KWIA 3 and 4 Rings, respectively. The zoomed insets illustrate SNR changes, while the subtracted images show residual noise patterns between the full dose and rest experimental conditions respectively. Similar to the simulated digital phantom study, a greater level of noise can be observed with real 60% and 30% dose scans, which were recovered to be comparable to that of the full dose scan with KWIA. There are residual edge signals in the subtracted images between low doses and full dose images, due to slight displacement of the phantom between scans. Nevertheless, the consistency of the residual noise pattern and edge signals across the low dose and KWIA reconstructed images suggests that KWIA does not introduce structured noise pattern or texture changes.

Table V lists the SNR and CNR measurements of the phantom images under seven experimental conditions respectively. The measured SNR and CNR values of low dose scans strictly follow the theoretical value predicted by Eq. 4, which validates our low dose simulation performed in section 3. B and 3. D. It can be seen that KWIA is able to recover SNR and CNR of low dose scans to be comparable with that of the full dose scan. The more rings used in KWIA, the greater SNR and CNR recovery.

**Fig. 5.**
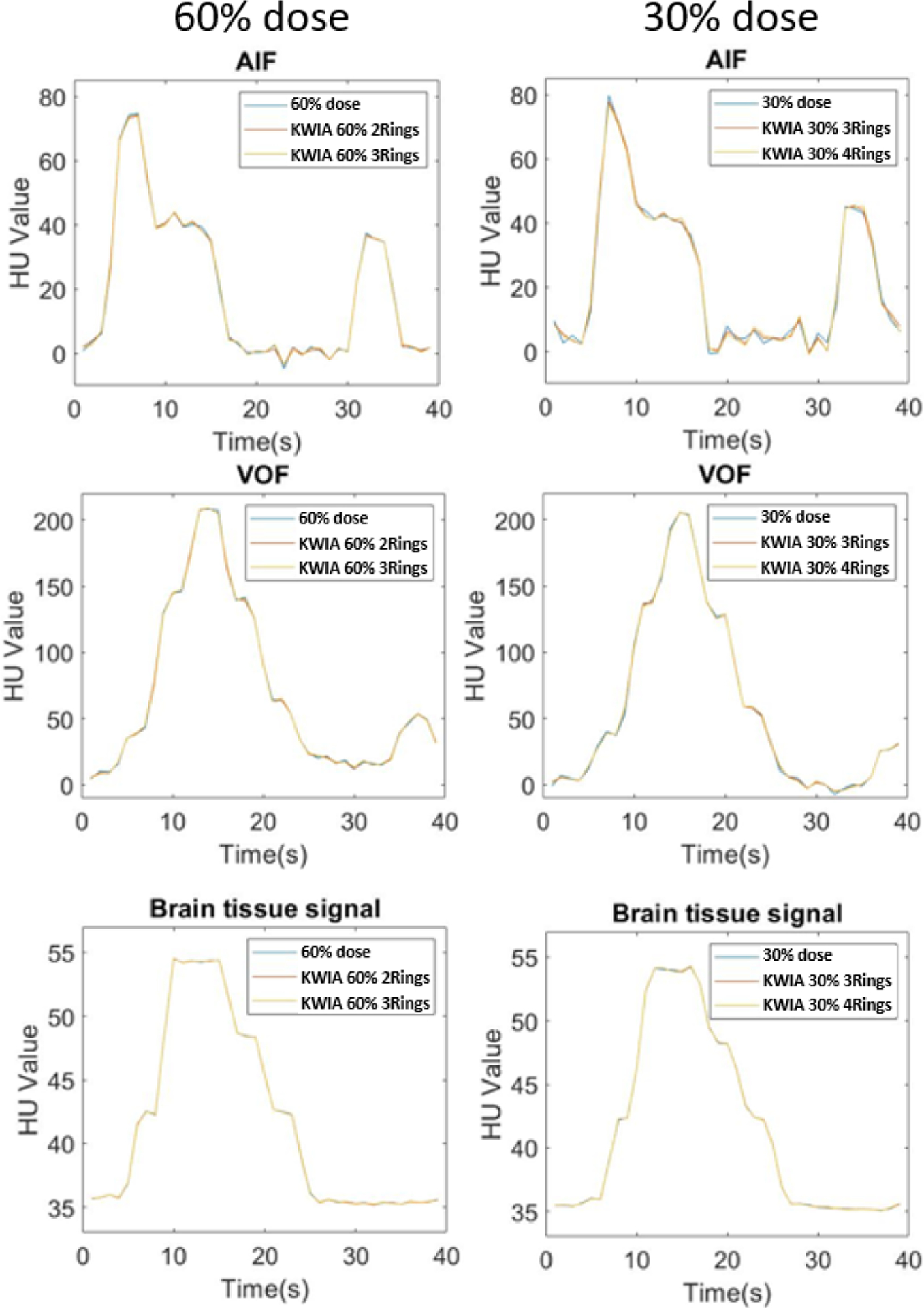
AIF, VOF and brain tissue signal curves of full dose and low dose images reconstructed with KWIA. No apparent difference can be observed all curves. The second peaks in AIF and VOF represent the second-pass of contrast bolus.

Figure 5 shows AIF, VOF and brain tissue signal curves. No apparent differences can be observed between time curves of full dose and low dose images reconstructed with KWIA suggesting that that no temporal blurring was introduced by KWIA.

**Fig. 6.**
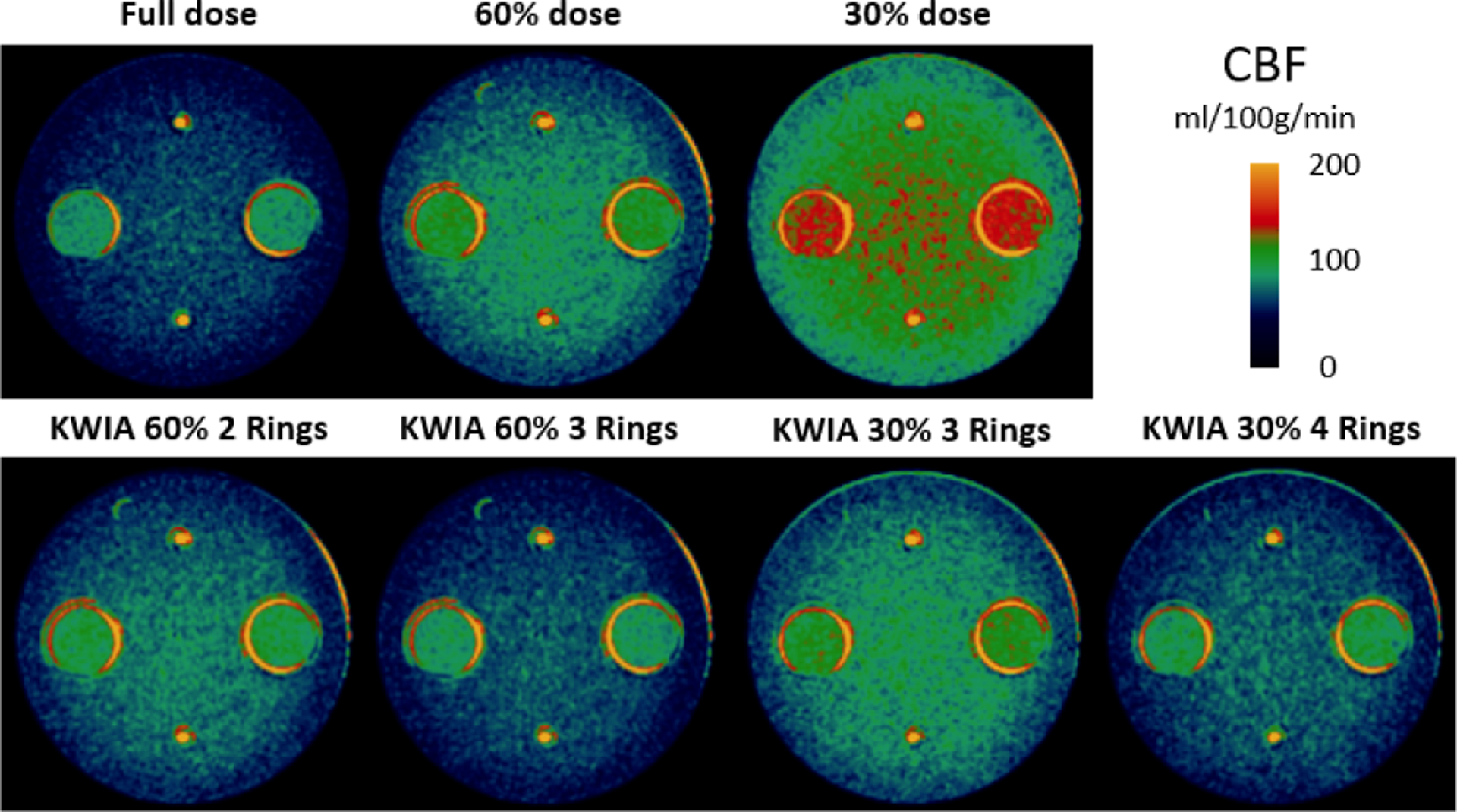
CBF maps (ml/100g/min) of the CTP phantom. Bias is introduced in the low dose CBF maps, which is corrected by KWIA.

**Fig. 7.**
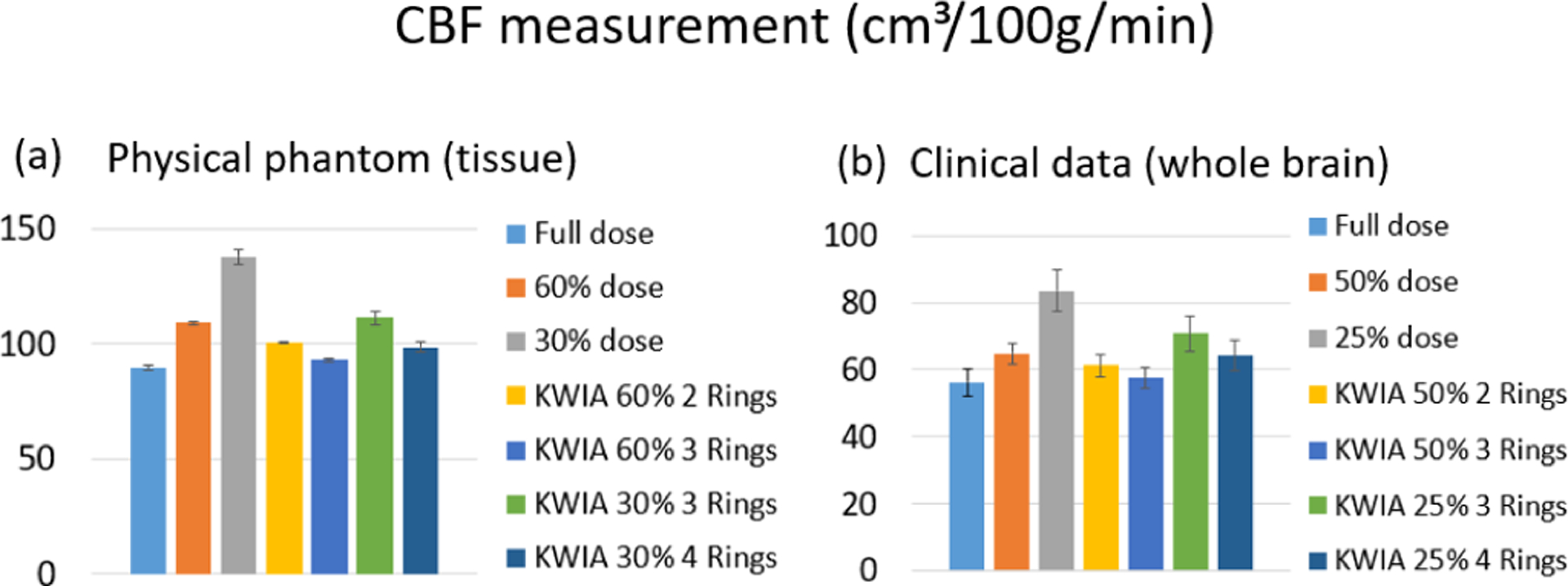
Bar plots of the mean CBF values for the physical phantom (a) and the clinical data (b) in full dose, low dose, and 4 KWIA conditions. For physical phantom, each condition includes 2 measurements in 2 tissue regions. And for the clinical data, each condition contains 6 whole brain measurements from 6 clinical datasets. Error bars indicate standard deviation.

Figure 6 shows quantitative CBF maps of the CTP phantom with seven different experimental conditions. It can be observed that an increasing bias was introduced in the CBF map with decreasing radiation dose (from full dose to 60% and 30% dose). The bias was corrected with KWIA reconstruction (see Fig. 7 (a)), and the resultant CBF maps were visually similar to that of the full dose scan.

### C. Clinical CTP Data Simulation

**Fig. 8.**
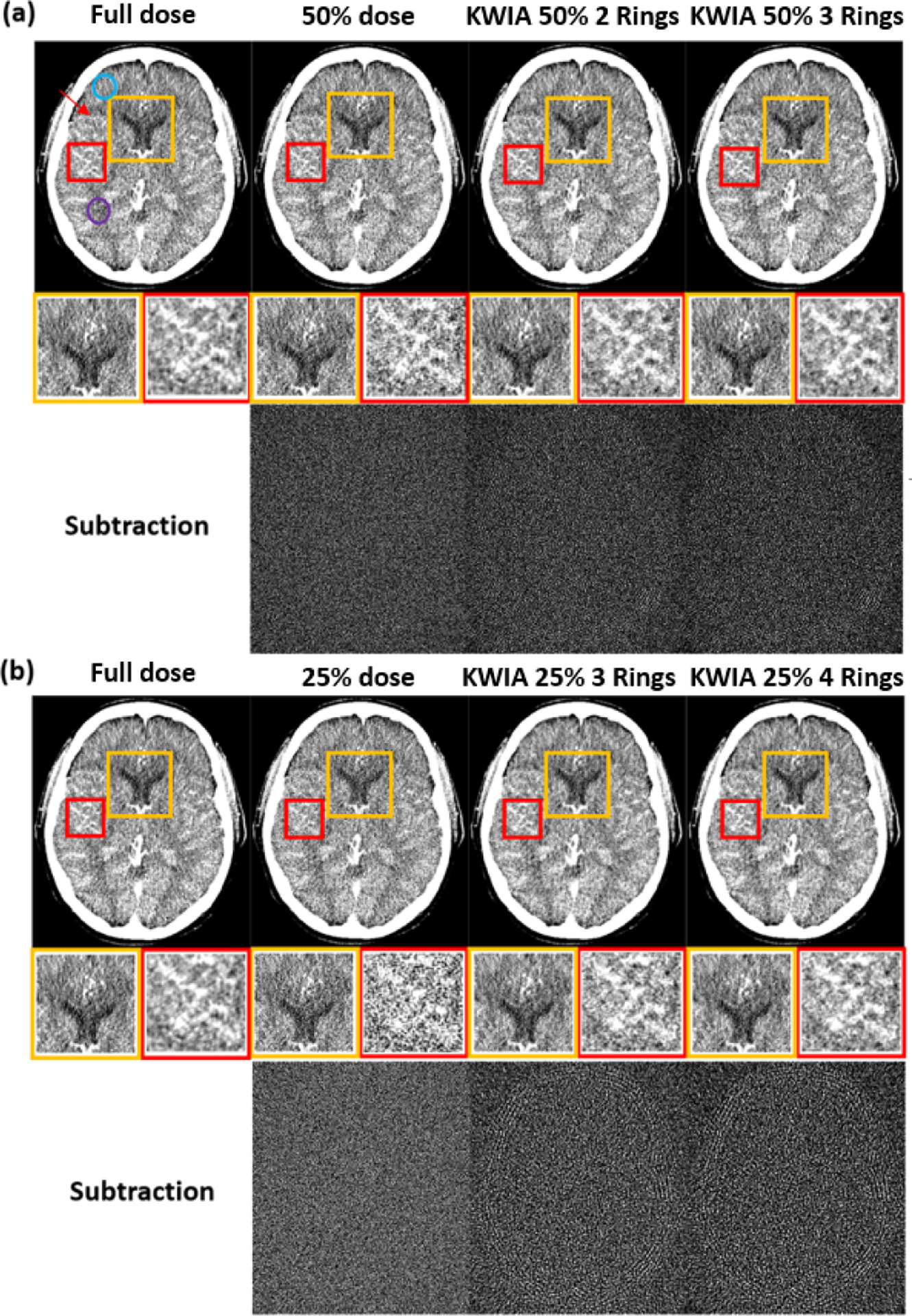
Clinical CT images, 25% and 50% dose simulation from original dose, and KWIA reconstructions. (a) and (b) contains the full dose, low dose simulation, and 4 KWIA reconstruction results. Two ROI were enlarged to emphasize SNR change. And subtraction images (window level and window center were adjusted for visual observation) were made to show the structural change. Visible SNR and CNR reduction can be observed in 50% and 25% dose simulation cases, and KWIA’s ability of SNR recovery can also be visually captured. In ROIs, the SNR changes can be seen more clearly, the performance of noise reduction in ROI 1 and contrast recovery in ROI 2 can be demonstrated with KWIA. No structural difference can be detected from subtraction images.

Figure 8 shows a representative image (15th time frame) of clinical CTP data, including simulated 50% and 25% doses and KWIA reconstructed images. The insets show two zoomed ROIs to better illustrate the SNR difference. The SNR reduction from full dose to 50% and 25% dose can be clearly observed, whereas this SNR reduction can be successfully recovered to be comparable to full dose level with KWIA reconstruction. The subtraction images between KWIA and full dose images show no structured noise pattern or texture changes introduced by KWIA reconstruction. Due to possible motion occurring between frames, some low-level ringing artifacts can be observed in subtraction images.

Table VI shows quantitative measurements of grey and white matter ROIs across the 7 experimental conditions. The SNR of simulated 50% dose images was 75% (WM) and 71% (GM) (70.7% in theory) of full dose SNR, and 54% (WM) and 50% (GM) (50% in theory) for 25% dose images. On 50% dose images, KWIA reconstruction with 2 and 3 rings improved SNR to 92% and 100% of full dose level respectively for white matter, and 89% and 95% respectively for grey matter. On 25% dose images, KWIA reconstruction with 3 and 4 rings improved SNR to 89% and 95% of full dose level respectively for white matter, and 74% and 84% for grey matter. As for noise SD, simulated 50% dose images increased SD to 1.38 (WM) and 1.37 (GM) (1.41 in theory) times of full dose level, and simulated 25% dose images increased SD to 1.84 (WM) and 2.05 (GM) (2 in theory) times of full dose level. KWIA reconstruction also decreased noise SD to full dose level. The CNR measured between WM and GM was 0.38 (0.39 in theory) for 50% dose and 0.28 (0.28 in theory) for 25% dose. KWIA also showed its ability to significantly improve CNR.

**Fig. 9.**
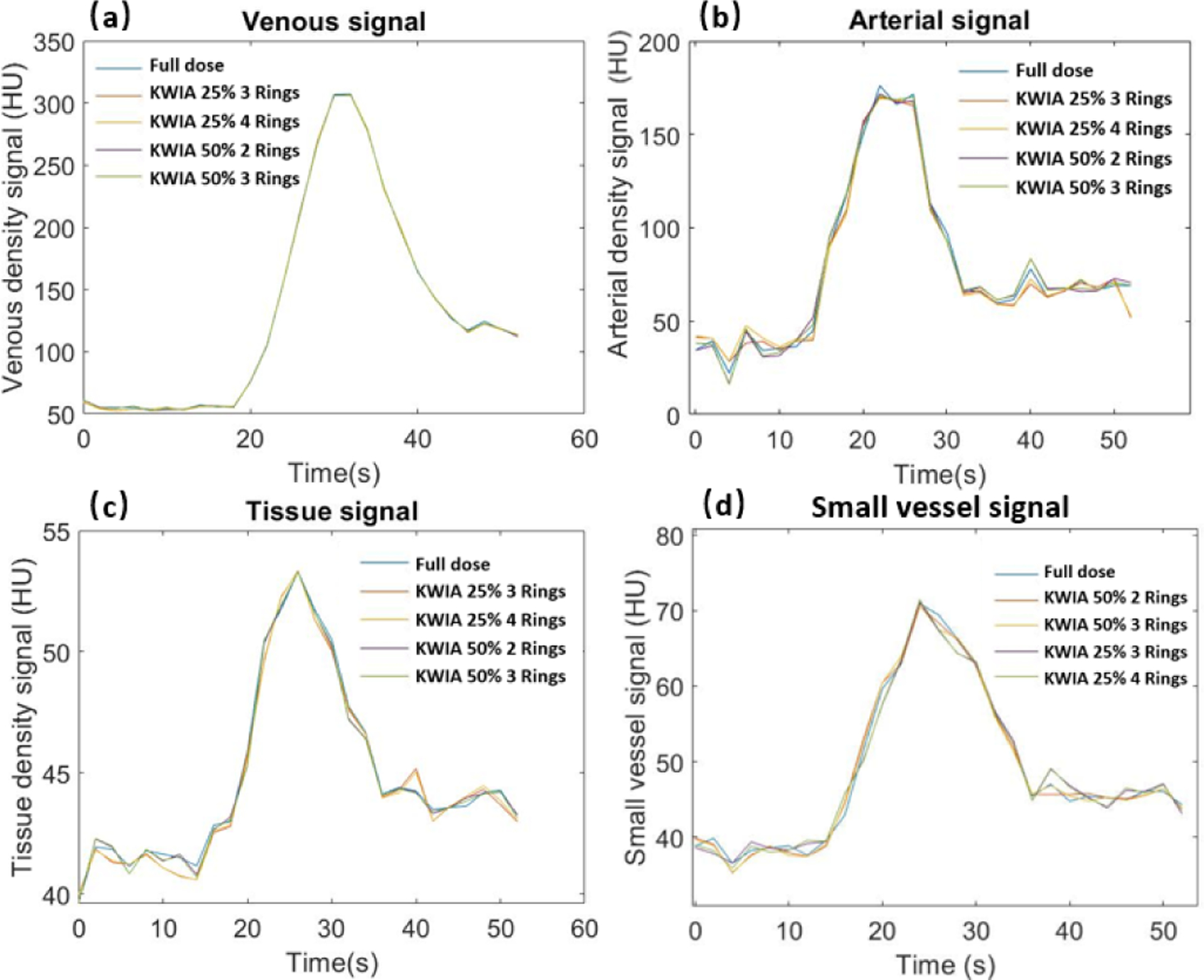
Dynamic contrast curves for venous (a), arterial (b), tissue ROI (c), and an about 1 mm wide small vessel (d) of full dose case and KWIA simulation cases. No apparent differences can be observed in all 4 signals. In arterial, tissue, and the small vessel signals, KWIA simulation with 25% dose reduction tends to have a greater difference than KWIA simulation with 50% dose reduction.

The VOF, AIF, and tissue signal curves of full dose and 4 KWIA reconstructions are presented in Fig. 9 (a), (b), and (c), respectively. The signal curves of 4 KWIA reconstructions closely follow those of the full dose images.

To evaluate the potential impact of KWIA on small vessels due to the averaging of high spatial frequency signals, Fig. 9 (d) shows the dynamic signal curves of a small vessel with a width about 1 mm. No apparent temporal smoothing was observed for this small vessel with KWIA reconstructions.

**Fig. 10.**
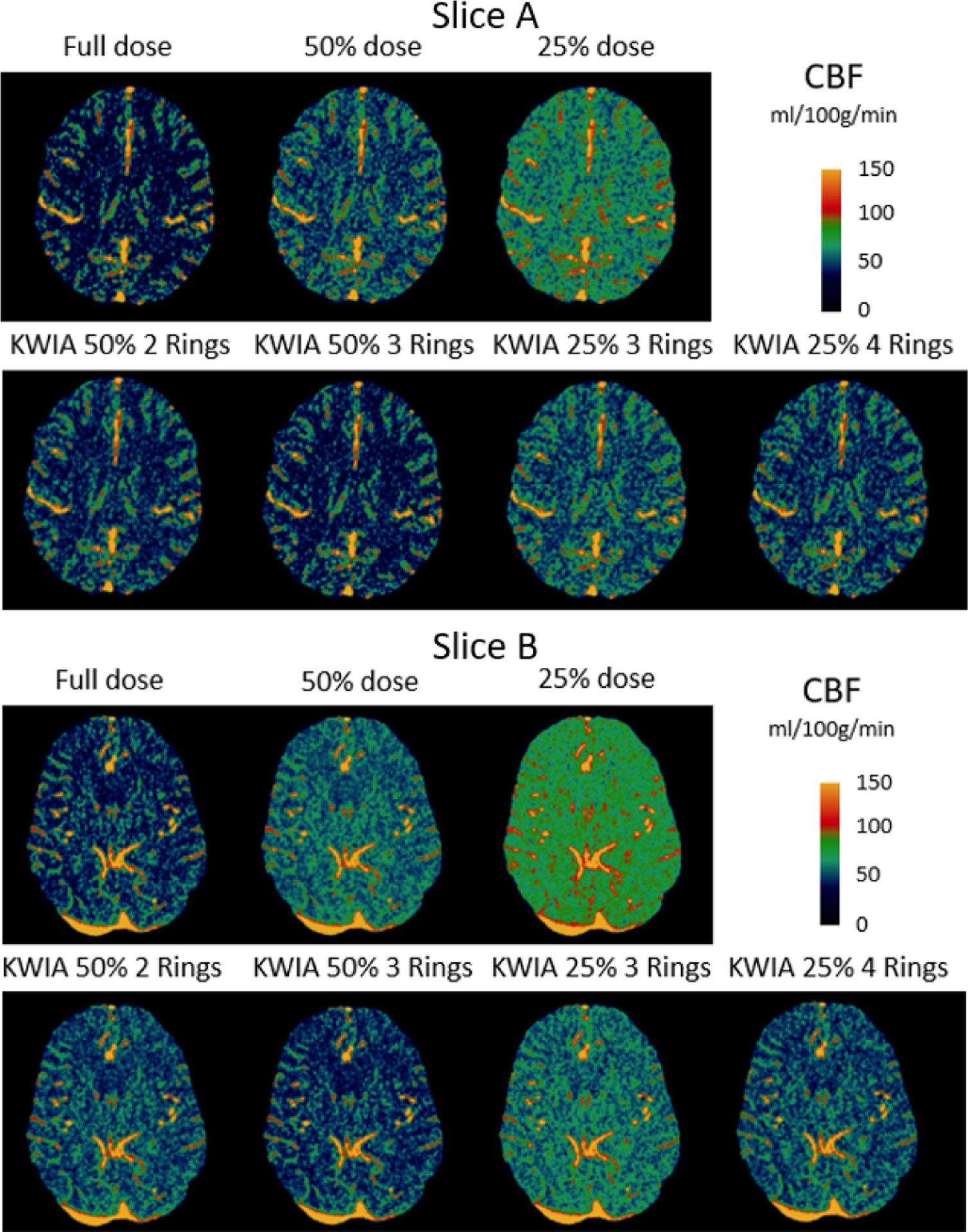
CBF maps (ml/100g/min) of 2 clinical CTP cases. From top to bottom are full dose, 50% and 25% dose with regridding reconstruction, as well as KWIA 50% with 2 and 3 Rings, KWIA 25% with 3 and 4 Rings respectively.

**Fig. 11.**
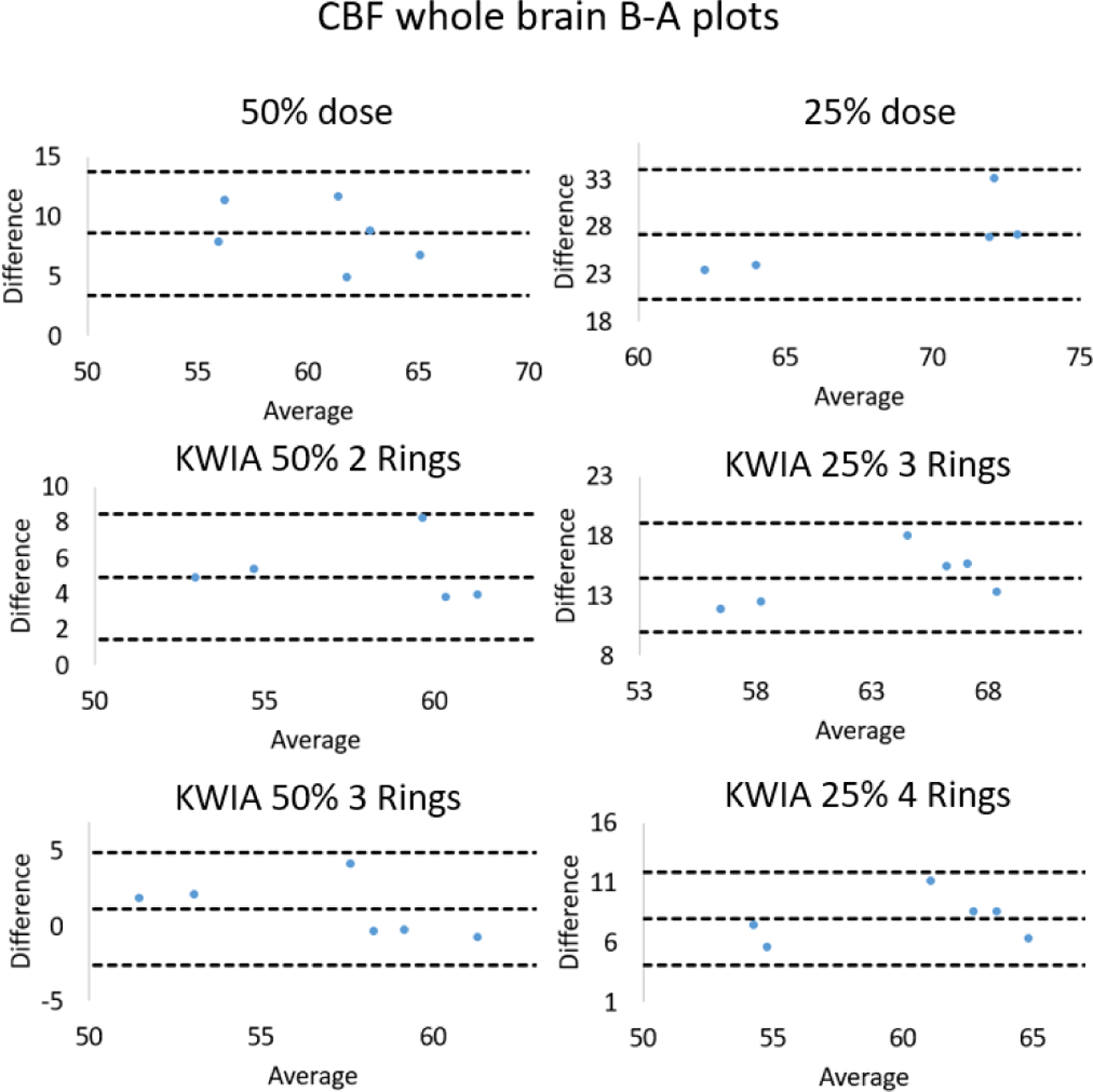
Bland-Altman plots for comparisons of whole brain CBF values measured between full dose and low dose conditions as well as low dose with KWIA reconstructions.

Quantitative CBF maps of a clinical case are shown in Fig. 10. Reduction of radiation dose to 50% and 25% introduced a substantial bias in the quantification of CBF maps, which was larger at 25% compared to 50% dose. However, the CBF maps of KWIA reconstructions were able to substantially correct the bias, especially in KWIA 50% 3 Rings and KWIA 25% 4 Rings which are visually comparable with the full dose images and show improved contrast between grey and white matter. The quantitative CBF values of the 7 conditions are displayed as bar plots in Fig. 7 (b). Finally, CBF values measured in whole brain between full dose and each low dose or KWIA case are demonstrated by the Bland-Altman plots in Fig. 11. CBF biases or mean differences were obvious in low dose conditions (8.6 and 27.2ml/100g/min for 50% and 25% respectively), which were minimized or reduced in all KWIA reconstructions. However, there was a small bias (~ 7ml/100g/min) between CBF values calculated with full dose and KWIA 25% 4 Rings CTP data. Low dose conditions also showed wider limits of agreement or 95% confidence interval of CBF differences (10.4 and 13.7 ml/100g/min for 50% and 25% dose respectively) compared with their corresponding KWIA reconstructions with the same dose (7.0 and 7.6 ml/100g/min for KWIA 50% with 2 and 3 rings, respectively, and 9.1 and 7.8 ml/100g/min for KWIA 25% with 3 and 4 Rings, respectively). The variability of scatters was consistent across the graph, suggesting that the change of difference does not depend on the average.

### D. Comparison With Other Reconstruction Algorithms

**Fig. 12.**
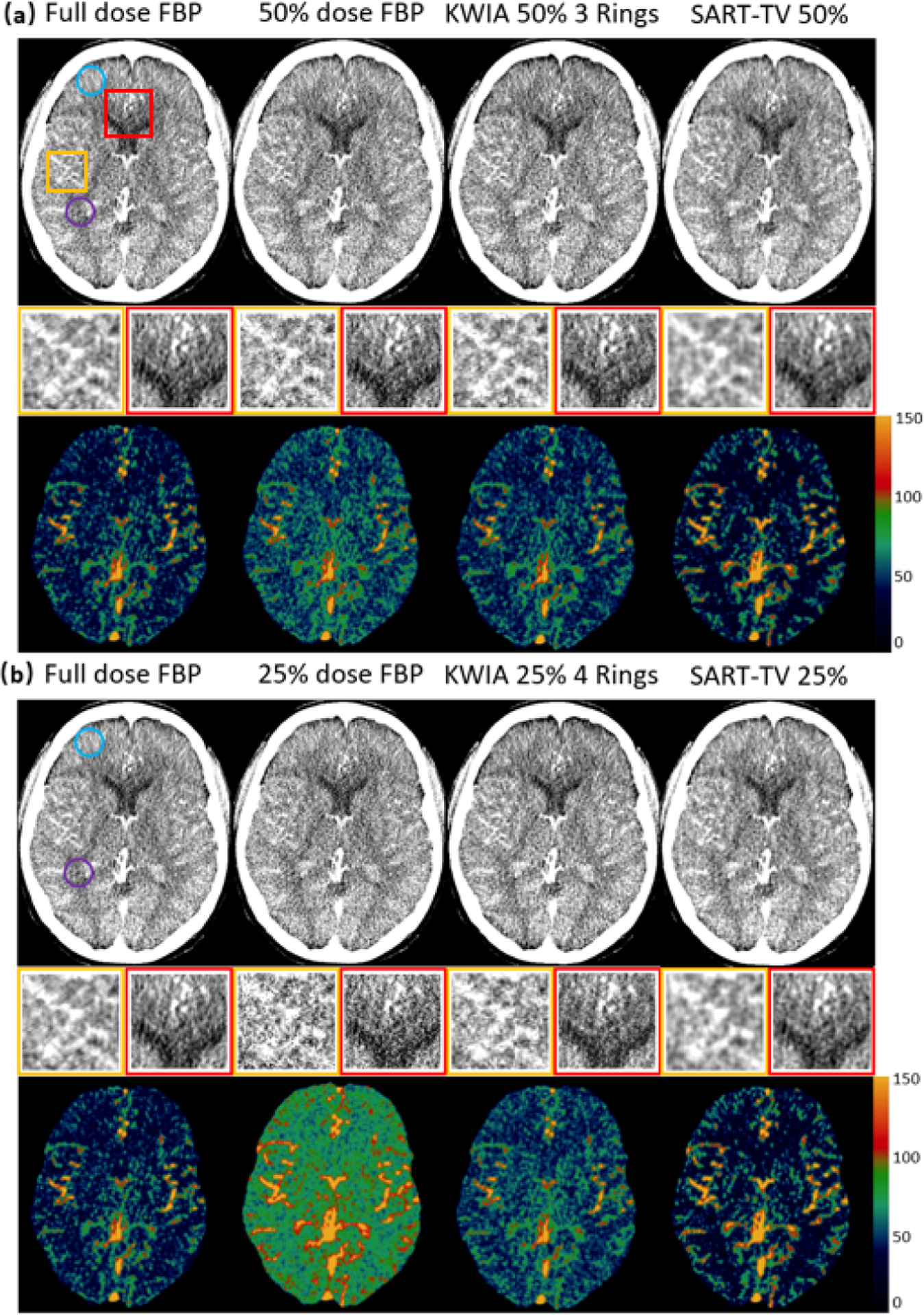
Reconstructed images (top row of (a) and (b)) and CBF maps (bottom row of (a) and (b)) with full dose FBP, 50% dose FBP, 25% dose FBP, KWIA 50% 3 Rings, KWIA 25% 4 Rings, 50% dose SART-TV, and 25% dose SART-TV. Insets with magnified regions of images show SNR and spatial smoothness of each reconstruction method.

Figure 12 displays the CTP images and CBF maps of full dose FBP (gold standard), 3 simulated 50% dose (Fig. 12 (a)) and 3 simulated 25% dose (Fig. 12 (b)) reconstructed by FBP, KWIA and SART-TV respectively. The SNR and CNR values for CTP images, and mean CBF values for CBF maps are listed in Table VII. The 50% and 25% dose FBP images exhibit large image degradation (26% (GM) and 20% (WM) SNR reduction for 50% dose, and 54% (GM) and 60% (WM) SNR reduction for 25% dose) and CBF overestimation (19% increase for 50% dose, and 48% increase for 25% dose), whereas KWIA yielded excellent reconstruction results comparable to the full dose FBP (92% (GM) and 96% (WM) of full dose FBP for 50% dose, and 82% (GM) and 88% (WM) of full dose FBP for 25% dose) without introducing smoothing effect or loss of spatial resolution. The CBF bias due to 50% and 25% dose, which were 10.6 and 27.2 ml/100g/min respectively, was largely suppressed by KWIA to 2.3 and 9.1 ml/100g/min respectively. SART-TV showed stronger denoising effect than KWIA with SNR and CNR higher than those of full dose FBP images. However, there was a slight over-correction of CBF bias using SART-TV (−4.3 and −1.4ml/100g/min for 50% and 25% respectively) and the reconstructed images appeared smoothed. It might be possible to achieve better performance of SART-TV by tuning hyperparameters to balance denoising power and spatial smoothness. However, the tuning process of SART-TV is constrained by the prolonged computation time, which further limits the use of SART-TV in clinical CTP scans.

Table VII also lists the execution time (ET) of reconstructing a 512-by-512 image from a 728-by-1152 sinogram using FBP, KWIA, and SART-TV respectively. With the same implementation environment (MATLAB, Intel i5–9400F), it took 11.2 seconds for KWIA to reconstruct an image which was similar to the reconstruction time of 9.3 seconds required by FBP. In comparison, SART-TV took 265.8 seconds with a graphic processing unit (MATLAB, GTX 1660 Ti).

## V. Discussion

Projection imaging data such as CT can be related to the spatial frequency domain (e.g., k-space in MRI) through the central-slice theorem by performing 1-D FT of each projection of an object, which is equivalent to a line through the center of the 2-D FT plane (i.e., k-space). By converting the CT sinogram into “k-space” data, we can adapt many innovative MRI reconstruction algorithms to preserve high spatial and temporal resolutions of undersampled CT data. In a previous study, we introduced an innovative image reconstruction algorithm based on k-space weighted image contrast (KWIC) [23], [41] for radiation dose reduction of CTP [24]. Our preliminary results showed that KWIC was able to reduce the radiation dose of existing CTP methods by 50–75% without compromising imaging speed or quality. However, the original KWIC algorithm requires rapid-switching pulsed X-ray at pre-specified rotation angles – a hardware capability not available on most commercial CT scanners.

In order to address this limitation, here we introduce a novel algorithm termed k-space weighted image average (KWIA) that preserves image quality (SNR and CNR), spatial and temporal resolutions, as well as quantification accuracy of low-dose CTP data (50–75% dose reduction) to be comparable to those of standard CTP scans. Unlike KWIC which requires a modified CT hardware, KWIA can be implemented by simply reducing the tube current. In this work, we demonstrated the feasibility of KWIA using both digital phantom and clinical CTP data with simulated low doses, as well as a physical CTP phantom with real low dose scans. Compared to existing low dose CT techniques such as iterative reconstruction, our approach is unique and has several advantages: 1) It is based on Fourier based CT image reconstruction, does not make assumptions of noise characteristics, and preserves the texture and resolution of CT images; 2) It has a low computational overhead and doesn’t affect the clinical workflow; and 3) It does not require modification of existing CT hardware, and therefore has a low barrier for clinical adoption. The k-space noise at different frequencies is averaged when converting into image space through FT [42], therefore KWIA improves the SNR of CTP images without affecting the resolution, texture or other characteristics. Previous studies have shown that the accuracy of CTP quantification is highly dependent on the noise level of CT images [43], [44]. Overestimation of perfusion often occurs in the presence of substantial noise using singular value decomposition (SVD) based deconvolution analysis. By recovering the SNR of low dose CTP images, KWIA was able to correct the bias of perfusion quantification with 50–75% dose reduction.

Only the applications of KWIA on 2D parallel beam and fan beam CT were evaluated in this study. Nevertheless, the theoretical principle of KWIA is applicable to low dose 3D cone beam CT (CBCT). Specifically, the central slice theorem for 3D CBCT geometry states that 1D FT of any 1D Radon data of a 3D object, which can be obtained indirectly with Grangeat’s method, is identical to the same radial line in the 3D k-space [45]. Across different time frames, KWIA will be able to partition, weight and average these radial lines from the center of the 3D k-space to the periphery, if a complete 3D Radon space can be obtained in CBCT. Previous studies have also shown the reliability and efficiency of Fourier based reconstruction for 3D CBCT [46]. Alternatively, for CBCT with circular geometry, where only the middle plane defined by the X-ray source trajectory has a complete set of Radon data, approximate reconstruction [47] can be applied on the projection data of the off-middle planes which can be converted to k-space for KWIA processing (with the caveat that the larger the cone angle, the less accurate the approximation is).

Despite KWIA’s potential to reduce CTP dose, it has a few limitations. KWIA improves image SNR by averaging high frequency k-space data with neighboring time frames, and is therefore potentially more sensitive to patient head motion than standard CTP scans. Another potential drawback of KWIA is the temporal blurring of dynamic signal changes of fine vessels and/or structures. As shown in Fig. 3, slight temporal blurring can be observed in 2.5 mm vessel of KWIA images, but not in vessels with 5 and 10mm sizes. Nevertheless, significant temporal blurring of clinical CTP data with KWIA reconstructions was not observed. The AIF and VOF curves reconstructed with KWIA also matched well with those of standard CTP data, and no apparent temporal signal deviations were observed for a vessel with ~1mm size. The potential temporal blurring of KWIA may depend on various parameters such as the rate of signal change and sampling rate of CTP, which merit further evaluation. In addition, iterative reconstruction algorithms such as SART-TV may have stronger denoising capability than KWIA. Nevertheless, KWIA is more advantageous in terms of the ease and robustness for implementation, computational speed, and retainment of texture and resolution. Comparison of KWIA with other iterative reconstruction and deep learning based denoising methods should also be performed in future studies. Lastly, the CBF bias reduction performance of KWIA was only evaluated by the CBF maps generated from standard SVD CTP analysis in this study. Alternative CTP analysis with denoising capabilities such as Bayesian probabilistic method need to be tested using KWIA reconstructed CTP images [48].

In this work, KWIA was applied to simulated low dose CTP data with reduced X-ray tube current which has a relatively straightforward relationship with SNR. It is also possible to reduce tube voltage, the square of which is generally acknowledged to be proportional to the received radiation dose [49]. The temporal window size or footprint of KWIA was kept as short as possible to minimize potential temporal blurring in this study. Nevertheless, the windowing function for averaging neighboring time frames as well as the number and size of rings in KWIA could be further optimized based on trade-offs between SNR improvement and the loss of temporal resolution. Alternative functions such as inverse NUFFT (iNUFFT) may be applied for regridding reconstruction. In the future, deep learning based approaches may be combined with KWIA to further improve its robustness in the presence of patient head motion or other artifacts (e.g. streaking due to photon starvation). Lastly, KWIA may be directly applied on CTP data acquired with standard radiation dose to reduce noise and enhance image contrast.

## VI. Conclusion

In this research, we presented a new low dose CTP technique termed KWIA, with a constant reduced tube current and projections averaging in outer k-space. The proposed technique was evaluated using a digital phantom, a physical phantom and clinical CTP data, and it can achieve considerable dose-savings (50–75%) without compromising the image quality and perfusion metrics. Due to its robustness and simplicity, KWIA may provide a promising method for reducing radiation exposure to patients undergoing CTP exams.

## Figures and Tables

**TABLE I:** Imaging and Reconstruction Parameters

Parameters	Digital phantom simulation	Physical phantom scan	Clinical CTP dataset simulation
Slices	1	3	4
Tube voltage (kVp)	NA	100	80
Tube current (mAs)	NA	200, 120, 60	100, 50, 25
Cycle time (s)	2	1	2
Cycles	27	39	27
Projections per turn	1152	1152	1152
Detectors	728	736	728
Slice thickness (mm)	NA	10	8
Pixel spacing(mm^2^)	0.75 × 0.75	0.59 × 0.59	0.48 × 0.48
Image size	512 × 512	512 × 512	512 × 512
Axial coverage (mm)	384	300	243.75
Source-to-detector (mm)	NA	1085.6	Unknown
Source-to-patient (mm)	NA	595	Unknown
CDTIvol (mGy)	NA	344,206,103	184,92,46

**TABLE II:** KWIA Ring Size (Radius) Definition

	Digital phantom & clinical data		Physical phantom	
	KWIA 50%	KWIA 25%	KWIA 60%	KWIA 30%
Ring	2 / 3 Rings	3 / 4 Rings	2 / 3 Rings	3 / 4 Rings
Ring 1	130 / 130	92 / 92	142 / 142	100 / 100
Ring 2	364 / 234	182 / 182	368 / 256	234 / 190
Ring 3	NA / 364	364 / 273	NA / 368	368 / 280
Ring 4	NA/NA	NA / 364	NA / NA	NA / 368

**TABLE III:** The Digital Phantom SNR and CNR Measurement in Different Conditions

Cases	Full dose	50% dose	25% dose	KWIA 50% 2Rings	KWIA 50% 3Rings	KWIA 25% 3Rings	KWIA 25% 4Rings
SNR	3.79	2.76	1.98	3.68	4.34	3.37	3.98
	/	73%	52%	97%	115%	89%	105%
CNR	0.85	0.61	0.43	0.81	0.95	0.75	0.89
	/	72%	51%	95%	112%	88%	105%

**TABLE IV:** RMSE, AUC, and FWHM Measurement for 3 Vessels With Different Size Contained in FORBILD Phantom

		Full dose	KWIA 50% 2Rings	KWIA 50% 3Rings	KWIA 25% 3Rings	KWIA 25% 4Rings
RMSE	2.5 mm	**/**	0.014	0.016	0.026	0.027
	5 mm	/	0.006	0.006	0.010	0.011
	10 mm	/	0.003	0.003	0.005	0.005
AUC	2.5 mm	10.2	10.1	10.1	10.1	10.1
	error	**/**	~1%	~1%	~1%	~1%
	5 mm	12.2	12.2	12.2	12.3	12.3
	error	**/**	<1%	<1%	~1%	~1%
	10 mm	13.1	13.1	13.1	13.1	13.1
	error	**/**	<1%	<1%	<1%	<1%
FWHM(s) 2.5 mm		13.7	12.7	12.7	12.9	13.0
	error	**/**	7%	7%	6%	5%
	5 mm	13.1	12.9	12.9	13.0	13.1
	error	**/**	2%	2%	~ 1%	<1%
	10 mm	12.9	13.0	13.0	12.9	12.9
	error	**/**	~1%	~1%	<1%	<1%

**TABLE V:** The Physical Phantom SNR and CNR Measurement in Different Conditions

Cases	Full dose	60% dose	30% dose	KWIA 60% 2Rings	KWIA 60% 3Rings	KWIA 30% 3Rings	KWIA 30% 4Rings
SNR	2.04	1.56	1.08	2.06	2.40	1.82	2.11
	/	76%	53%	101%	118%	89%	103%
CNR	1.77	1.36	0.89	1.83	2.11	1.47	1.72
	/	77%	50%	103%	119%	83%	97%

**TABLE VI:** Quantitative Measurement of SNR and CNR in Clinical Data

Cases	Full dose	50% dose	25% dose	KWIA 50% 2 Rings	KWIA 50% 3 Rings	KWIA 25% 3 Rings	KWIA 25% 4 Rings
Mean(GM)	52.2	52.3	52.8	52.3	52.3	52.7	52.8
Mean(WM)	40.3	41.0	41.0	41.0	41.0	41.3	41.4
SD(GM)	13.9	19.1	28.4	15.6	14.4	18.8	16.5
SD(WM)	16.8	23.2	31.0	18.4	17.2	20.6	18.5
SNR(GM)	3.8	2.7	1.9	3.4	3.6	2.8	3.2
	/	71%	50%	89%	95%	74%	84%
SNR(WM)	2.4	1.8	1.3	2.2	2.4	2.0	2.2
	/	75%	54%	92%	100%	83%	92%
CNR	0.6	0.4	0.3	0.5	0.5	0.4	0.5
	/	67%	50%	83%	83%	67%	83%

**TABLE VII:** Quantitative Comparison Among FBP, KWIA, and SART-TV

Cases	Full dose FBP	50% dose FBP	25% dose FBP	KWIA 50% 3 Rings	KWIA 25% 4 Rings	50% dose SART- TV	25% dose SART- TV
SNR(GM)	3.9	2.9	2.1	3.6	3.2	5.5	4.5
SNR(WM)	2.5	2	1.5	2.4	2.2	3.7	3.28
CNR	0.6	0.4	0.3	0.5	0.5	0.8	0.6
CBF(WB)	55.1	65.7	81.3	57.4	64.2	50.8	53.7
	±2.8	±4.6	±6.3	±3.4	±5.0	±2.6	±2.6
ET(s)	9.3	9.3	9.3	11.2	11.2	265.8	265.8

## References

[1] Powers WJ , “2018 Guidelines for the early management of patients with acute ischemic stroke: A guideline for healthcare professionals from the American heart association/American stroke association,” Stroke, vol. 49, no. 3, pp. e46–e99, 2018. 29367334 10.1161/STR.0000000000000158

[2] Wintermark M , “Acute stroke imaging research roadmap II,” Stroke, vol. 44, no. 9, pp. 2628–2639, 2013. 23860298 10.1161/STROKEAHA.113.002015 PMC4040226

[3] Campbell BCV , “Endovascular therapy for ischemic stroke with perfusion-imaging selection,” New England J. Med, vol. 372, no. 11, pp. 1009–1018, 2015. 25671797 10.1056/NEJMoa1414792

[4] Saver JL , “Stent-retriever thrombectomy after intravenous t-PA vs. T-PA alone in stroke,” New England J. Med, vol. 372, no. 24, pp. 2285–2295, 6 2015. 25882376 10.1056/NEJMoa1415061

[5] Krishnan P, Murphy A, and Aviv RI, “CT-based techniques for brain perfusion,” Topics Magn. Reson. Imag, vol. 26, no. 3, pp. 113–119, 2017. 10.1097/RMR.0000000000000129 28520657

[6] Abels B, Klotz E, Tomandl BF, Villablanca JP, Kloska SP, and Lell MM, “CT perfusion in acute ischemic stroke: A comparison of 2-second and 1-second temporal resolution,” Amer. J. Neuroradiol, vol. 32, no. 9, pp. 1632–1639, 10 2011. 21816919 10.3174/ajnr.A2576 PMC7965399

[7] Othman AE , “Radiation dose reduction in perfusion CT imaging of the brain: A review of the literature,” J. Neuroradiol, vol. 43, no. 1, pp. 1–5, 2 2016. 26452610 10.1016/j.neurad.2015.06.003

[8] Lee T-Y and Chhem RK, “Impact of new technologies on dose reduction in CT,” Eur. J. Radiol, vol. 76, no. 1, pp. 28–35, 10 2010. 20643522 10.1016/j.ejrad.2010.06.036

[9] Yu Z, Thibault J-B, Bouman CA, Sauer KD, and Hsieh J, “Fast model-based X-ray CT reconstruction using spatially nonhomogeneous ICD optimization,” IEEE Trans. Image Process, vol. 20, no. 1, pp. 161–175, 1 2011. 20643609 10.1109/TIP.2010.2058811

[10] Beister M, Kolditz D, and Kalender WA, “Iterative reconstruction methods in X-ray CT,” Phys. Medica, vol. 28, no. 2, pp. 94–108, 4 2012. 10.1016/j.ejmp.2012.01.003 22316498

[11] Geyer LL , “State of the art: Iterative CT reconstruction techniques,” Radiology, vol. 276, no. 2, pp. 339–357, 8 2015. 26203706 10.1148/radiol.2015132766

[12] Xiao Y , “STIR-Net: Deep spatial-temporal image restoration net for radiation reduction in CT perfusion,” Frontiers Neurol, vol. 10, pp. 1–16, 6 2019. 10.3389/fneur.2019.00647 PMC6607281 31297079

[13] Kang E, Min J, and Ye JC, “A deep convolutional neural network using directional wavelets for low-dose X-ray CT reconstruction,” Med. Phys, vol. 44, no. 10, pp. e360–e375, 10 2017. 29027238 10.1002/mp.12344

[14] Chen H , “Low-dose CT with a residual encoder-decoder convolutional neural network,” IEEE Trans. Med. Imag, vol. 36, no. 12, pp. 2524–2535, 12 2017. 10.1109/TMI.2017.2715284 PMC5727581 28622671

[15] Chen H , “LEARN: Learned experts’ assessment-based reconstruction network for sparse-data CT,” IEEE Trans. Med. Imag, vol. 37, no. 6, pp. 1333–1347, 6 2018. 10.1109/TMI.2018.2805692 PMC6019143 29870363

[16] Yang Q , “Low-dose CT image denoising using a generative adversarial network with wasserstein distance and perceptual loss,” IEEE Trans. Med. Imag, vol. 37, no. 6, pp. 1348–1357, 6 2018. 10.1109/TMI.2018.2827462 PMC6021013 29870364

[17] Chen H , “Low-dose CT via convolutional neural network,” Biomed. Opt. Express, vol. 8, no. 2, pp. 679–694, 2017. 28270976 10.1364/BOE.8.000679 PMC5330597

[18] Wolterink JM, Leiner T, Viergever MA, and Išgum I, “Generative adversarial networks for noise reduction in low-dose CT,” IEEE Trans. Med. Imag, vol. 36, no. 12, pp. 2536–2545, 12 2017. 10.1109/TMI.2017.2708987 28574346

[19] Wu D, Kim K, El Fakhri G, and Li Q, “Iterative low-dose CT reconstruction with priors trained by artificial neural network,” IEEE Trans. Med. Imag, vol. 36, no. 12, pp. 2479–2486, 12 2017. 10.1109/TMI.2017.2753138 PMC5897914 28922116

[20] Kang E, Chang W, Yoo J, and Ye JC, “Deep convolutional framelet denosing for low-dose CT via wavelet residual network,” IEEE Trans. Med. Imag, vol. 37, no. 6, pp. 1358–1369, 6 2018. 10.1109/TMI.2018.2823756 29870365

[21] Gupta H, Jin KH, Nguyen HQ, McCann MT, and Unser M, “CNN-based projected gradient descent for consistent CT image reconstruction,” IEEE Trans. Med. Imag, vol. 37, no. 6, pp. 1440–1453, 6 2018. 10.1109/TMI.2018.2832656 29870372

[22] Wang Y , “Iterative quality enhancement via residual-artifact learning networks for low-dose CT,” Phys. Med. Biol, vol. 63, no. 21, 10 2018, Art. no. 215004. 10.1088/1361-6560/aae511 30265251

[23] Song HK and Dougherty L, “Dynamic MRI with projection reconstruction and KWIC processing for simultaneous high spatial and temporal resolution,” Magn. Reson. Med, vol. 52, no. 4, pp. 815–824, 2004. 15389936 10.1002/mrm.20237

[24] Martin T, Hoffman J, Alger JR, McNitt-Gray M, and Wang DJ, “Low-dose CT perfusion with projection view sharing,” Med. Phys, vol. 45, no. 1, pp. 101–113, 1 2018. 29080274 10.1002/mp.12640 PMC5774228

[25] Primak AN, McCollough CH, Bruesewitz MR, Zhang J, and Fletcher JG, “Relationship between noise, dose, and pitch in cardiac multi–detector row CT,” Radiographics, vol. 26, no. 6, pp. 1785–1794, 11 2006. 17102050 10.1148/rg.266065063

[26] van Aarle W , “Fast and flexible X-ray tomography using the ASTRA toolbox,” Opt. Express, vol. 24, no. 22, pp. 25129–25147, 2016. 27828452 10.1364/OE.24.025129

[27] van Aarle W , “The ASTRA toolbox: A platform for advanced algorithm development in electron tomography,” Ultramicroscopy, vol. 157, pp. 35–47, 10 2015. 26057688 10.1016/j.ultramic.2015.05.002

[28] Malik WQ, Khan HA, Edwards DJ, and Stevens CJ, “A gridding algorithm for efficient density compensation of arbitrarily sampled Fourier-domain data,” in Proc. IEEE/Sarnoff Symp. Adv. Wired Wireless Commun, 2005, pp. 125–128.

[29] Jackson JI, Meyer CH, Nishimura DG, and Macovski A, “Selection of a convolution function for Fourier inversion using gridding (computerised tomography application),” IEEE Trans. Med. Imag, vol. 10, no. 3, pp. 473–478, 9 1991. 10.1109/42.97598 18222850

[30] Yu Z, Noo F, Dennerlein F, Wunderlich A, Lauritsch G, and Hornegger J, “Simulation tools for two-dimensional experiments in X-ray computed tomography using the FORBILD head phantom,” Phys. Med. Biol, vol. 57, no. 13, pp. N237–N252, 7 2012. 22713335 10.1088/0031-9155/57/13/N237 PMC3426508

[31] Benner T, Heiland S, Erb G, Forsting M, and Sartor K, “Accuracy of gamma-variate fits to concentration-time curves from dynamic susceptibility-contrast enhanced MRI: Influence of time resolution, maximal signal drop and signal-to-noise,” Magn. Reson. Imag, vol. 15, no. 3, pp. 307–317, 1 1997. 10.1016/s0730-725x(96)00392-x 9201678

[32] Mayo JR , “Simulated dose reduction in conventional chest CT: Validation study,” Radiology, vol. 202, no. 2, pp. 453–457, 2 1997. 9015073 10.1148/radiology.202.2.9015073

[33] Amir O, Braunstein D, and Altman A, “Dose optimization tool,” Proc. SPIE, vol. 5029, pp. 815–821, 5 2003.

[34] Frush DP , “Computer-simulated radiation dose reduction for abdominal multidetector CT of pediatric patients,” Amer. J. Roentgenol, vol. 179, no. 5, pp. 1107–1113, 11 2002. 12388482 10.2214/ajr.179.5.1791107

[35] Žabić S, Wang Q, Morton T, and Brown KM, “A low dose simulation tool for CT systems with energy integrating detectors,” Med. Phys, vol. 40, no. 3, 2 2013, Art. no. 031102. 10.1118/1.4789628 23464282

[36] Sun Nuclear Corporation. CT Perfusion Phantom. Accessed: Mar. 18, 2020 [Online]. Available: https://www.sunnuclear.com/products/ct-perfusion-phantom

[37] Østergaard L, Weisskoff RM, Chesler DA, Gyldensted C, and Rosen BR, “High resolution measurement of cerebral blood flow using intravascular tracer bolus passages. Part I: Mathematical approach and statistical analysis,” Magn. Reson. Med, vol. 36, no. 5, pp. 715–725, 11 1996. 8916022 10.1002/mrm.1910360510

[38] Benson TM and De Man BKB, “Synthetic CT noise emulation in the raw data domain,” in Proc. IEEE Nucl. Sci. Symp. Med. Imag. Conf, 10 2010, pp. 3169–3171.

[39] Jia X, Lou Y, Li R, Song WY, and Jiang SB, “GPU-based fast cone beam CT reconstruction from undersampled and noisy projection data via total variation,” Med. Phys, vol. 37, no. 4, pp. 1757–1760, 3 2010. 20443497 10.1118/1.3371691

[40] Biguri A, Dosanjh M, Hancock S, and Soleimani M, “TIGRE: A MATLAB-GPU toolbox for CBCT image reconstruction,” Biomed. Phys. Eng. Express, vol. 2, no. 5, 9 2016, Art. no. 055010.

[41] Song HK and Dougherty L, “K-space weighted image contrast (KWIC) for contrast manipulation in projection reconstruction MRI,” Magn. Reson. Med., Off. J. Int. Soc. Magn. Reson. Med, vol. 44, no. 6, pp. 825–832, 2000. 10.1002/1522-2594(200012)44:6<825::aid-mrm2>3.0.co;2-d 11108618

[42] Brown RW, Cheng Y-CN, Haacke EM, Thompson MR, and Venkatesan R, Magnetic Resonance Imaging: Physical Principles and Sequence Design. Hoboken, NJ, USA: Wiley, 2014.

[43] Manniesing R, Oei MTH, van Ginneken B, and Prokop M, “Quantitative dose dependency analysis of whole-brain CT perfusion imaging,” Radiology, vol. 278, no. 1, pp. 190–197, 1 2016. 26114226 10.1148/radiol.2015142230

[44] Li K and Chen G-H, “Dependence of quantitative accuracy of CT perfusion imaging on system parameters,” Proc. SPIE, vol. 10132, 3 2017, Art. no. 101320D.

[45] Buzug TM, Computed Tomography: From Photon Statistics to Modern Cone-Beam CT. Berlin, Germany: Springer, 2008.

[46] Schaller S, Flohr T, and Steffen P, “An efficient Fourier method for 3-D radon inversion in exact cone-beam CT reconstruction,” IEEE Trans. Med. Imag, vol. 17, no. 2, pp. 244–250, 4 1998. 10.1109/42.700736 9688156

[47] Feldkamp LA, Davis LC, and Kress JW, “Practical cone-beam algorithm,” J. Opt. Soc. Amer. A, Opt. Image Sci, vol. 1, no. 6, pp. 612–619, 1984.

[48] Boutelier T, Kudo K, Pautot F, and Sasaki M, “Bayesian hemodynamic parameter estimation by bolus tracking perfusion weighted imaging,” IEEE Trans. Med. Imag, vol. 31, no. 7, pp. 1381–1395, 7 2012. 10.1109/TMI.2012.2189890 22410325

[49] Seyal AR, Arslanoglu A, Abboud SF, Sahin A, Horowitz JM, and Yaghmai V, “CT of the abdomen with reduced tube voltage in adults: A practical approach,” Radiographics, vol. 35, no. 7, pp. 1922–1939, 11 2015. 26473536 10.1148/rg.2015150048

